# Sensors to Increase the Security of Underwater Communication Cables: A Review of Underwater Monitoring Sensors

**DOI:** 10.3390/s20030737

**Published:** 2020-01-29

**Authors:** Dimitrios Eleftherakis, Raul Vicen-Bueno

**Affiliations:** Research Department, NATO STO Center for Maritime Research and Experimentation (CMRE), 19100 La Spezia, Italy; Dimitrios.Eleftherakis@cmre.nato.int

**Keywords:** Underwater, Communication cables, Sensors, Surveillance, Monitoring, Detection, Marine objects

## Abstract

Underwater communication cables transport large amounts of sensitive information between countries. This fact converts these cables into a critical infrastructure that must be protected. Monitoring the underwater cable environment is rare and any intervention is usually driven by cable faults. In the last few years, several reports raised issues about possible future malicious attacks on such cables. The main objective of this operational research and analysis (ORA) paper is to present an overview of different commercial and already available marine sensor technologies (acoustic, optic, magnetic and oceanographic) that could be used for autonomous monitoring of the underwater cable environment. These sensors could be mounted on different autonomous platforms, such as unmanned surface vehicles (USVs) or autonomous underwater vehicles (AUVs). This paper analyses a multi-threat sabotage scenario where surveying a transatlantic cable of 13,000 km, (reaching water depths up to 4000 m) is necessary. The potential underwater threats identified for such a scenario are: divers, anchors, fishing trawls, submarines, remotely operated vehicles (ROVs) and AUVs. The paper discusses the capabilities of the identified sensors to detect such identified threats for the scenario under study. It also presents ideas on the construction of periodic and permanent surveillance networks. Research study and results are focused on providing useful information to decision-makers in charge of designing surveillance capabilities to secure underwater communication cables.

## 1. Introduction: Underwater Communication Cables, a Critical Infrastructure to Protect

Underwater communication cables are the dominant way of transporting information. They account for 97% of the world’s data traffic, while satellites account for less than 3% [1]. The advantages of fiber-optic cables compared to satellites include higher reliability and capacity, lower transmission delay, lower cost and higher bandwidths. Services include money transfers, transfers of sensitive information (e.g., political and military), etc. Therefore, underwater communication cables are of strategic importance to global social welfare. This make them a critical infrastructure that should be protected by all means.

**Figure 1:**
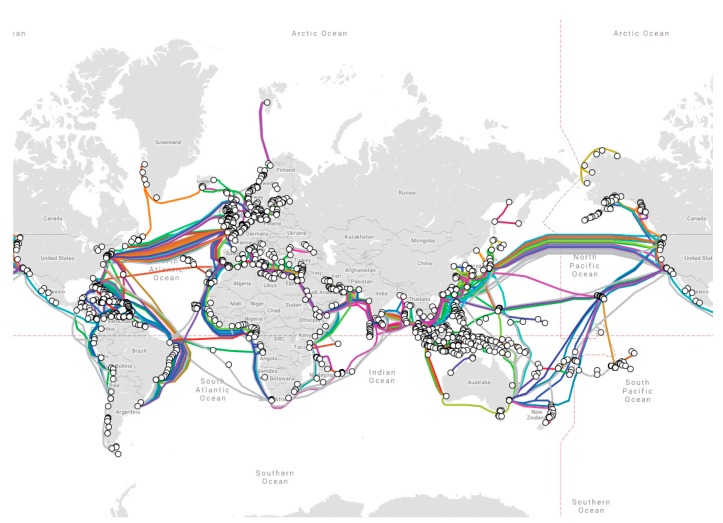
Distribution of underwater communication cables on Earth [2]. The cable routes are stylized and do not reflect the actual path taken by the systems. Screenshot generously provided by TeleGeoGraphy [2].

The average cable system capacity increased from 20 Tbps in 2014 to 60 Tbps in 2017, reaching up to 208 Tbps (MAREA cable from USA to Spain) in 2019. As of early 2019, there were approximately 378 submarine cables in service around the world, making a total of about 1.2 million of kilometers of cable [2]. Nevertheless, the total number of cables is constantly changing, as new cables enter into service and older cables are decommissioned. The distribution of underwater communication cables in the world can be seen in Figure 1.

**Figure 2:**
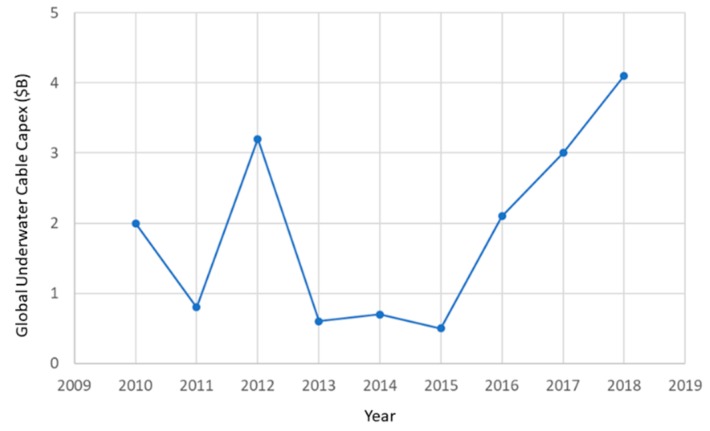
Approximate global underwater cable construction investment between 2010 and 2018 [3].

The capital expenditure on underwater cables from 2010 till 2018 is shown in Figure 2 [3]. Approximately 50 new projects have been proposed (to date) for the next three years (2019–2021), representing a total investment of 7.2 billion dollars. About 30% of the expected deployments will be in the Pacific region, 21% in the Atlantic region and 17% in the Indian Ocean. Present investments are driven mainly by large web-based and telecommunication companies [3]. Demand growth is built into cable deployments. Most underwater cables are designed with a significant buffer between total capacity and actual capacity. On average, 15 to 30% of underwater cable capacity is lit, allowing for large spikes in demand to be accommodated (by rerouting traffic), if, for example, a cable would be damaged [3]. The life expectancy of an underwater cable is estimated at 25 years.

The main conventions that protect underwater cables are:

- 1884: International Convention for the Protection of Submarine Cables [4];
- 1958: The Geneva Conventions of the Continental Shelf and High Seas [5];
- 1982: United Nations Convention on Law of the Sea (UNCLOS) [6].

The latest (UNCLOS) establishes the rights and duties of all states, balancing the interests of coastal states in offshore zones with the interests of all states in using the oceans. UNCLOS treats all cables the same, no matter whether they are used for telecommunications or power transmission or for commercial, military or scientific purposes. The aforementioned conventions established a framework that tries to balance competing uses and interests. Its success lies on its interpretation and implementation by the different parties, but it has not met the original expectations so far [7].

The underwater cables are subject to a number of threats such as: Natural (e.g., earthquakes, volcano or tsunami); Accidental (e.g., fishing, anchor dragging or dredging); and Malicious (e.g., theft or terrorism). Despite these threats, many states have not taken protection measures to enhance the security of these cables. In addition, penalties are not imposed on offenders for negligent or deliberate damage.

Some organizations and countries are trying to address these threats in various ways depending on the individual challenges they are facing. The main forum in which issues about the submarine cable protection are discussed is the International Cable Protection Committee Ltd. (ICPC) [8]. It has over 175 members representing telecommunication and power companies, government agencies and scientific organizations from more than 60 countries. Its role is extremely important as it continuously monitors the situation regarding underwater cables around the world, it promotes cooperation between the different users and it issues recommendations regarding methods for the protection of underwater cables. There are also Regional Cable Protection Committees (RCPCs) [7], such as: the Subsea Cables UK, the North American Submarine Cables Association (NASCA), the Oceanic Submarine Cable Association (OSCA) and the Danish Cable Protection Committee (DKCPC).

New Zealand, Australia, Uruguay, and Colombia [7] are adopting cable protection legislation. In 1996 [9], the New Zealand government passed the Submarine Cables and Pipelines Protection Act for controlling fishing and anchoring along cable route locations, resulting in the formation of cable protection zones. In July 2007, the Australian Communication and Media Authority (the ACMA) made two declarations for underwater cable protection zones off the Sydney coast [10]. The zones extend from shore to 2000 m water depth, with ranges from 56.9 km to 75 km from shore. The widths of the zones range from 4.4 km (close to the shore) to 15.7 km (at 2000 m depth) [7].

Despite the efforts being made to ensure the integrity of the cables, the measures taken are mostly sporadic. Indeed, monitoring of the underwater environment where the cables lie is rare, and any intervention/maintenance usually follows cable faults. The necessity to regularly monitor the underwater area of the cables is highlighted in several reports during the last few years, raising issues about possible future malicious attacks on the cables (e.g., [11]).

Based on the identified risks and the need to survey the underwater cable areas to protect them, this paper focuses on providing the results of an investigation done regarding the different commercial, already available, sensors and platforms that could potentially be used for monitoring/surveying the underwater environment of a cable route at regular intervals. The identified sensors use different operation principles, and most of them could be mounted in different autonomous platforms.

This operational research and analysis (ORA) research paper is organized as follows. Section 2 describes the underwater environment where the cables lay, the regulations regarding cable deployment and the potential threats they are exposed to. Section 3 provides an overview of the research results regarding monitoring sensors and unmanned platforms that could carry the identified sensors. Section 4 provides the results for a case study and the design requirements for a surveillance capability based on the identified sensors and platforms. Finally, Section 5 summarizes the main conclusions, and provides recommendations on possible future work. 

To conclude this introduction, it is worth mentioning that the aforementioned topics drive the paper to provide basic knowledge to final users (underwater cable surveillance network developers) on several options existing for constructing intelligence, surveillance and reconnaissance (ISR) protection networks for underwater/submarine cables in general, and for underwater communication cables in particular. This has not been provided in the open literature, as far as the authors know. This foundation could be used in future more-detailed and specific studies on actually designing security measures.

## 2. The Underwater Cable Environment and the Identification of Threats

Section 2.1 describes the basic characteristics of underwater cable design and provides a description on their installation procedure on the seabed. Section 2.2 provides details on the states’ jurisdictions over seawaters. Section 2.3 presents an analysis of the different threats to which cables are exposed to in the underwater domain. Finally, Section 2.4 provides a synthesis of the underwater environment in terms of water depth and distance (range) from the shore.

### 2.1. Underwater Cable Environment: Communication Cable Specifications and Seabed Installation

Modern underwater communication cables use fiber-optic technology. The cable diameter depends on the water depth. In shallow waters, the cables are double armored and the diameter is around 46 mm. The protection becomes lighter as the water depth increases, resulting in smaller cable diameter. The range of cable diameter with respect to the water depth is summarized in Table 1. The armored version consists of optical fibers surrounded by: (a) supporting core, (b) wire (for strength), (c) copper conductor (for powering the repeaters), and (d) polyethylene dielectric case. Wire armor is used for additional protection. The maximum deployment depth can reach up to 8000 m for the light-weight cable version (see Table 1). The breaking load of the same cable type is approximately 90 kN [12]. Note that repeaters are used (usually every 70 km) to amplify the optical signals.

Prior to the installation, the optimal cable route has to be determined. The main considerations that impact the route selection process are: (a) geopolitical constraints, (b) military operation areas, (c) water depth, (d) seabed lithology, (e) seabed slopes, (f) seabed stability (currents, tides), (g) existence of volcanoes or tectonic plates, (h) fishing activities, (i) wrecks, and (j) unexploded ordnance (UXO).

In critical continental shelf areas with high marine activity (e.g., trawl fishing, ship anchoring), cable burial is required. The cables are buried beneath the seabed using sea plows or remotely operated vehicles (ROVs, in operations deeper than 1000 m). The typical cable burial depth is 1.0–1.5 m, but it can extend up to 3 m in softer seabed [7]. Burial may typically extend from the shore to about 1000–1500 m water depths [13,14], conditioned to suitable seabed morphology. In the cases where the cable cannot be buried due to hard seabed, articulated or (split) pipes offer additional protection. In areas of strong currents, articulated pipes can also be secured with saddle clamps/pins to the seabed at regular intervals so that the cable/line is secured in position [15]. 

The longest submarine communication cable is the SEA–ME–WE3 (South-East Asia–Middle East–Western Europe 3) cable. It reaches a length of 39,000 Km [16]. It has 39 landing points linking the aforementioned regions. This shows the installation complexity it presents.

### 2.2. Underwater Cable Environment: States’ Jurisdictions over Sea Waters

UNCLOS has established different zones of juridical competence for coastal states and other users of the sea. The different rights for the different sea users in the main zones are as follows:

- *Territorial Sea* (TS). It is the area that extends up to 12 nm (22 km) from the baseline of a country’s coast. The coastal State has sovereignty over it. Therefore, it can control ships engaged in cable operations and it can ask for specific requirements to be met (e.g., environmental) before giving permission to perform cable operations. Foreign ships (merchant and military) are allowed passage through the territorial sea. However, the coastal state may suspend the right to the passage if there is a threat to its security.
- *Contiguous Zone* (CZ). It extends 12 nm (22 km) beyond the TS limit. A coastal state has limited control and can take action against practices that violate its customs, fiscal, and immigration laws.
- *Legal Continental Shelf* (LCS)/*Exclusive Economic Zone* (EEZ). LCS extends up to a maximum of 350 nm (648 km) from the country’s coast and EEZ up to 200 nm (370 km) from its coast (if LCS is lower than 200 nm). The coastal state has sovereign rights to explore and exploit its natural resources inside this zone. UNCLOS (Article 58) affirms that all States have the freedom to lay submarine cables in the EEZ and LCS. Although not explicitly stated, the article includes the right to survey, repair and maintain underwater cables. The LCS is different from the *Geological Continental Shelf* (GCS) [7]. The average width of the GCS is about 43 nm (80 km) and the depth range is 120–200 m. At the maximum depth, GCS steepens onto the continental slope, and reaches depths of 1500–3500 m at an average inclination of (usually) 4° [7].
- *High Seas* (HS). High seas extent further away than the CS or EEZ limits. Article 112 of UNCLOS states that “*all States are entitled to lay (implying also survey, repair and maintenance) submarine cables and pipelines on the bed of the high seas beyond the continental shelf*”. High seas are open to all states for freedom of navigation, freedom of over flight, freedom to construct artificial island installations, freedom of fishing, and freedom of scientific research.

It has to be noted that underwater cables, unlike vessels, are not registered to any nationality. Since consortia or private companies (owning and operating them) are from different countries, one cable can span many different jurisdictions [14]. A thorough analysis of the topic covered in this sub-section can be found in [7].

### 2.3. Identification of Threats

**Figure 3:**
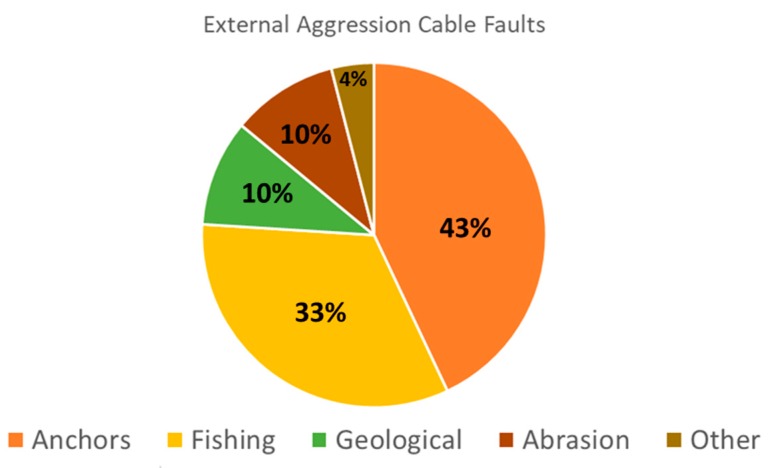
Underwater cable threats by category: comparison of cable faults from 2007 to 2018 [17].

Even with shielding and burying, more than 200 underwater cable faults happen every year on average [17]. Periodically, three underwater cable operators (SubCom, Alcatel Submarine Network, and Global Marine Systems Ltd.) publish statistics on cable faults based on their individual datasets. Figure 3 is based on their last report [17], which presents approximate percentages of the different causes of cable faults between 2007 and 2018. It is observed that anchors (43%) and fishing (33%) account for the majority (more than 75%) of the faults. Geological and abrasion causes account for 10% each, while the remaining 4% is attributed to other causes.

**Figure 4:**
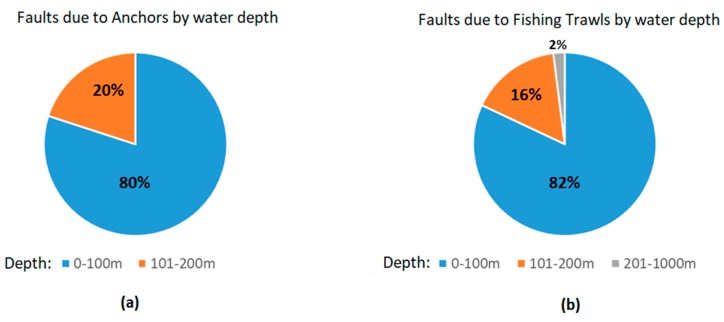
Underwater cable threats by water depth from 2016–2018: (**a**) anchor faults depth distribution, (**b**) fishing trawl faults depth distribution [17].

Figure 4a presents anchor faults by water depth during the last three years (2016–2018), accounting for most of them (~80%) in shallow waters (depth <100 m). Figure 4b presents fishing faults with respect to water depth for the same period, with similar results for shallow waters. During the last years, a slight decrease has been observed in the fishing faults in the 100–200 m range due to deeper burial depths (3 m, wherever possible) [17]. It also seems that fishing faults in the 300–800 m range have slightly increased, probably due to fishing activity moving into deeper waters [17].

In this study, the threats provoking cable faults are categorized as: natural hazard threats and man-made threats. More details in the following subsections.

#### 2.3.1. Natural Hazards

Natural hazards include: submarine earthquakes, landslides, currents and waves, tsunami storm surge, sea level raise, extreme weather (hurricanes), and volcanic activities. Several effects happen in cables and installations due to these natural threats, such as: abrasion, stress, fatigue, and failure.

The GCS face hazards due to weather and shelf/slope current systems. Storms cause either erosion of the seabed (waves) or large volume sediment transport, constituting a significant hazard. For example, Typhoon Morakot [7] struck Taiwan from 7 to 11 August 2009, accompanied with extremely heavy rain. This caused rivers to flood and carry vast amounts of sediments to the ocean, resulting in several seabed cable breaks. Tsunamis and earthquakes are less frequent in the GCS, but if they happen, their effect is catastrophic.

In general, the installation of a new cable system is site-specific due to the variable nature of natural hazards.

#### 2.3.2. Man-Made Threats

In this study, man-made threats are divided in accidental and intentional man-made threats.

Accidental man-made threats include man-made actions that can cause unintentionally cable faults, such as fishing trawls or anchors. There are different types of fishing trawls used in the fishing industry, most of them being able to damage underwater cables. Clam dredges can dig into the seabed up to 0.2 m, while scallop dredges scour the bottom. Both gear types are mainly used in shallow waters and can cause damage to insufficiently buried cables. Otter and beam trawls scour the seabed for harvesting bottom fish. Stow nets are used in tidal flows, where large anchors are utilized to slow their movement. As an example, such anchors have caused damage to even well-buried cables off the coast of China [7]. Anchor-provoked cable damages occur either due to ships anchoring outside approved anchorages, or due to anchor dragging by a vessel underway.

Most of the cable faults occur due to negligence from fishing and shipping activities. However, there are intentional man-made incidents provoked by human beings acting deliberately to damage cables. As an example: on 23 March 2007, two cable faults were detected: one in the TVH (Thailand–Vietnam–Hong Kong, length: 3354 km) fiber-optic underwater cable system, and another in the APCN (Asian Pacific Cable Network, length: 12,083 km) system; 98 km and 79 km of cable were stolen from TVH and ACPN, respectively. Estimates of damage to the two systems exceed USD 7.2 M [18]. It was proven that fishermen (probably deliberately) misunderstood the decision of the Vietnam government to allow them salvage aging copper cables (deployed before 1975), as they salvaged all but one of the country’s underwater cable links regardless of cable age [18]. Another relevant example: in 2013, the South East Asia–Middle East–Western Europe 4 (SEA–ME–WE 4) cable was intentionally cut by divers, crippling Internet speeds by 60% in Egypt [7]. Until today, there has been no major terrorist attack targeting underwater cables. However there is a growing amount of reports suggesting that underwater cables could be destroyed by terrorist groups [7], or tapped to gain access to the data transferred [19]. In December 2010, the US State Department strongly disapproved a WikiLeaks list of critical infrastructures, which included underwater cable information, providing terrorists a target list [20]. 

### 2.4. Synthesis of Threats with Respect to Cable Design/Installation, State Responsibility, Distance from the Shore and Water Depth 

**Figure 5:**
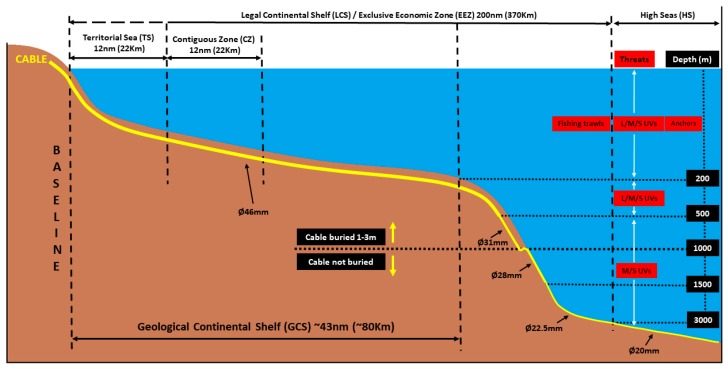
Schematic synthesis (not linearly scaled) including the most likely identified threats and the different components of the underwater cable environment (Ø = cable diameter). L/M/S UVs means large/medium/small underwater vehicles.

Figure 5 presents a schematic synthesis of the different components (covered in Section 2.1, Section 2.2 and Section 2.3) that comprise the underwater environment where underwater cables are installed. In more detail, the components that are shown are: the different cable diameters by water depth, the water depth ranges at which the cable is supposed to be buried, the different maritime zones, and the most prevalent threats.

## 3. Platforms and Sensors to Survey Underwater Communication Cables

This section presents the results of a study into the different types of underwater sensors that could potentially be used for surveying the underwater environment where the cables lay. It is important to note that for the construction of an underwater surveillance network, the sensors are linked to the platforms on which they are installed. For this reason, this section starts with Section 3.1, which presents an overview of the specifications of the identified platforms, mainly unmanned/autonomous. An important specification of the platforms is the type of monitoring sensors that they can carry. It continues with Section 3.2, which presents the detailed specifications of the identified sensors, sorted by kind. 

### 3.1. Platforms to Carry Sensors to Survey the Underwater Environment of Communication Cables

The performed study has identified different platforms that could be used to carry underwater sensors to survey the underwater environment of the communication cables, such as:

- Buoys, moorings and seafloor bottom mounts
  Moorings are usually used as instrumentation platforms for data collection. They are composed of three main parts: an anchor; a chain or line to attach instruments; and a flotation device. The flotation devices could be buoys, which could also be used as instrumentation holding platforms. Furthermore, instead of using a simple anchor, seafloor bottom mounts are also used to attach the mooring line to the seabed, which could be used as individual instrumentation platforms. Since the focus of the current paper is on platforms able to cover large distances where the cables lay, the specifications of commercial moorings are not discussed here. This kind of static platform is suitable to monitoring strategic locations along the cables.
- Unmanned Surface Vessels (USVs)
  USVs are vehicles that operate (autonomously or remotely operated, ROVs) on the sea-surface without a crew. Table 2 presents the most relevant specifications of some commercial USVs that could be used to carry the identified sensors to survey the underwater environment of the cables.
- Autonomous Underwater Vehicles (AUVs)
  AUVs are mobile platforms that are normally used for ocean survey operations. They are untethered vehicles and computer-controlled, with little or no operator interaction while performing their mission. They are more stable than towed platforms. They can collect data with more accuracy and reliability. A detailed study about AUVs was undertaken in [21] in 2015. Table 3 presents the main AUVs identified from this study, including their main characteristics towards facilitating surveillance network designers the selection of the required platform to carry the needed underwater sensors to survey the underwater environment of the cables.
- Unmanned Underwater Gliders (UUGs)
  A special type of AUV is the UUG. UUGs use changes in buoyancy rather than a propeller for underwater moving/navigation (although they can incorporate a propeller in the hybrid buoyancy-propelled versions). They have long endurance and long navigation range, but their payload capacity (especially with power demanding sensors) is limited. They can operate on the sea surface or underwater. When being underwater, they follow an up-and-down / saw-tooth-like profile. Table 4 presents the specifications of some commercial UUGs identified in the market.

Additional information about the aforementioned platforms can be found in [22]. A global inventory of AUVs and UUGs available in the commercial market can be found in [23].

Table 2, Table 3 and Table 4 present the main specifications of the selected USVs, AUVs, and UUGs available on the market. The most favorable values provided by the manufacturers in the specification brochures have been selected to compose the tables. It has to be noted that the name of each company in the tables appears in the same way shown in the website of each company. 

The general terms and abbreviations used in the tables are:

- N/P: Not Provided;
- N/A: Not Applicable;
- ASW: Anti-Submarine Warfare;
- Acoustic sensors on-board the platforms: Single-Beam Echo-Sounder (SBES), Side Scan Sonar (SSS), Synthetic Aperture Sonar (SAS), Multi-Beam Echo-Sounder (MBES), Sub-Bottom Profiler (SBP), Obstacle Avoidance Sonar (OAS), Diver Detection Sonar (DDS), Variable Depth Sonar (VDS), Passive sonars—Towed Array Sonar (TAS), Passive/Active sonars—Hull-Mounted Sonar (HMS), Passive sensors—Hydrophones, Dipping Sonar, Acoustic Doppler Current Profiler (ADCP), Altimeter;
- Magnetic (Magnet) sensors on-board the platforms: Magnetometers/Gradiometers;
- Optical sensors on-board the platforms: Cameras (CAM): Video Cameras (vCAM), Still Cameras (sCAM);
- Oceanographic (Ocean) sensors on-board the platforms: Conductivity, Temperature, Pressure, Sound velocity, Salinity, Turbidity, pH.

### 3.2. Sensors to Survey the Underwater Environment of Communication Cables

This section presents the different sensors identified in this study that could potentially be used to monitor the underwater environment where the communication cables are located. The sensors are categorized and discussed in the following subsections as follows:

- Acoustic (Section 3.2.1);
- Magnetic (Section 3.2.2);
- Optical (Section 3.2.3);
- Oceanographic (Section 3.2.4).

#### 3.2.1. Acoustic Sensors

Since electromagnetic waves are attenuated extremely fast in the water, acoustic waves turn into the best and most commonly used way for exploring and monitoring the underwater environment. This subsection briefly presents a number of identified sensors the operation principles of which are based on acoustics. Detailed information about some of these and other systems can be found in [24]. 

##### 3.2.1.1. Single-Beam Echo-Sounder (SBES)

The single-beam echo-sounder (SBES) sends an acoustic signal in the vertical direction (towards sea bottom) below the platform where installed. The sounder measures depth, which relates to the signal’s two-way travel time. It is also possible to extract information of the seabed type from the analysis of the echo [25]. Although the main use of SBES is to produce bathymetry maps, its capability to detect echoes from targets in the water column has turned it into a valuable tool for fishing.

The SBES beam has an angular aperture of approximately 5–15°. For achieving higher resolution in fishery sounders, multiple secondary beams are created by dividing the transducer’s transmitting beam into several sectors. This interferometry process is called split-beam. 

The coverage of the seafloor when using SBES is limited. A way to overcome it consists of installing several SBES on a traverse in order to increase the number of sounding points. This system is called Sweep sounder [26].

The motivation for considering SBES in this study is the capability of its fishery version to detect small targets along the water column and at the sea bottom. Therefore, it could potentially be used for detecting probable intruders in the cable environment. 

Table 5 presents the specifications of some selected commercial SBES. It includes common bathymetry SBES, fishery SBES, and a Swath system.

##### 3.2.1.2. Multi-Beam Echo-Sounder (MBES)

The multi-beam echo-sounder (MBES) is an extension of SBES. Nowadays it dominates hydrography due to its high spatial seabed coverage (usually 100%) at relatively short times. MBES transmits and receives a set (fan) of beams with small individual widths across the platform axis. MBES can measure simultaneously the water depth and the seabed reflectivity (backscatter). MBES provides the seafloor relief from depth measurements. MBES also provides seabed properties from the backscatter signal, being proven to be a reliable indicator of the sediment type. The finite dimension of the beams places a limit on the minimum target size that can be successfully detected [27,28,29].

MBESs are routinely used for mapping the seafloor of an area for selecting the optimum route for the installation of underwater cables on the seabed [7,30,31]. 

The motivation for considering MBES in this study is twofold: (a) it has the capability to detect objects on the seabed and along the water column; and (b) it can be used to map the seabed where the cables lay, in a fast way and with high resolution.

Table 6 presents the main specifications of some identified commercial MBESs.

##### 3.2.1.3. Side-Scan Sonar (SSS)

The side-scan sonar (SSS) produces acoustic images by ensonifying the seabed using two side antennas of very narrow horizontal directivity. SSS records the backscattered signal at grazing angles of incidence. It is usually installed on a platform (fish) towed close to the seabed. The traditional side scan sonar cannot measure bottom relief, but only gives a rough estimate of its altitude from the echo at nadir. It is however possible to compensate this effect by adding interferometric capabilities.

High-resolution SSS is commonly used for the detection of mines [32]. SSS is also routinely used for identifying the presence of obstacles on the seafloor to select the optimal route when deploying underwater cables [7,33]. SSS is also used for tracking underwater cables and pipelines after their installation [34].

Based on the work mentioned above, SSS can be used either for monitoring the status of an underwater cable or for surveying the underwater environment (including seabed) around the cable against objects/intruders that may harm the cables.

Table 7 presents the main specifications of some identified commercial SSSs.

##### 3.2.1.4. Synthetic Aperture Sonar (SAS)

Classical beam-forming has two main limitations: the spatial resolution is not homogeneous, and the resolution may become too low with increasing range. The synthetic aperture sonar (SAS) tries to address these limitations and improve the along-track resolution. The principle of SAS is to ensonify with several pings the same seabed area while the platform carrying the SAS is moving. The synthetic array is formed by the pings that have the image position within the beam-width. The coherent reorganization and combination of the signals produces a synthetic aperture image with improved along-track resolution compared to the conventional SSS.

SAS can be applied both for acoustic imaging and bathymetry (lower resolution). The main limitation of SAS is that all data corrections have to be applied almost perfectly, otherwise the performance decrease will be high. High-resolution SAS is used for detecting mines [35]. It can also be used to detect underwater communication cables that lay on the seabed [36]. 

As happened for SSS, SAS can be used either for monitoring the status of an underwater cable or for surveying the underwater environment (including seabed) around the cable against objects / intruders that may harm the cables.

Table 8 presents the main specifications of some identified commercial SASs.

##### 3.2.1.5. Sub-Bottom Profiler (SBP)

The sub-bottom profiler (SBP) operates in low frequencies. Its signal can penetrate the seabed and give an image of the upper seabed layers. Its working principle is not based on the backscatter (like the sonars used for seabed mapping, e.g., MBES). It is based on the reflection of a specular echo instead. SBP records echoes from the interfaces between the sediment layers below the seabed. At each interface, one part of the incident energy is reflected towards the surface, while the other part is transmitted to deeper layers.

Since conventional SBPs do not always achieve good lateral resolution, a parametric array of SBPs is commonly used, especially for the detection of shallow buried objects (e.g., pipelines, cables, mines). Its operation is based on non-linear acoustic techniques for achieving a narrow directivity pattern at low frequency. This has two advantages: (a) wide bandwidth; and (b) frequency independent beam pattern.

SBPs are used to map the sediments layers when determining the cable route and especially in the case where cable burial is required [7]. Focusing on this case of study, the surveillance of the underwater environment where the cable lies, SBPs can be used to detect anomalies/changes in the bottom properties affecting buried underwater communication cables [37].

Table 9 presents the main specifications of some identified commercial SBPs. 

##### 3.2.1.6. Obstacle Avoidance Sonar (OAS)

The obstacle avoidance sonar (OAS) is typically installed on submarines, ROVs or AUVs. They are normally focused on detection and identification of obstacles that may exist in front of them within a distance of a few tens or hundreds of meters. OAS operates at high underwater acoustic frequencies, horizontally scanning over a wide angular sector. The 3D versions of OAS are often called acoustic cameras. The multi-beam scanning in these systems is performed in two perpendicular planes.

Focusing on this case of study, OAS can be used for detecting targets in the underwater environment, such as underwater vehicles, which could pose a threat to underwater cables.

Table 10 presents the main specifications of some identified commercial OASs.

##### 3.2.1.7. Diver Detection Sonar (DDS)

The diver detection sonar (DDS) is based on the high-resolution “imaging” principle of MBES (see Section 3.2.1.2), with an extended horizontal field of view of 360°. Besides divers, DDS can also detect UUVs (size similar or larger than a diver).

The DDS is an important sensor in surveillance networks in ports and ships. Therefore, they can play the same role for monitoring the underwater communication cable areas for detecting divers and UUVs.

Table 11 presents the main specifications of some identified commercial DDSs.

##### 3.2.1.8. Variable Depth Sonar (VDS)

When a sonar operates near the water surface, it is usual to observe shadow zones in deep depths. This is due to strong signal reflections in the water surface that mask these zones. The variable depth sonar (VDS) works under the principle of installing the sonar array in a ‘fish’ towed at variable depths under the thermocline in order to compensate the detection range and to fill the gaps created by the shadow zones.

The VDS is used for detecting submarines, which can be a potential threat to underwater cables.

Table 12 presents the main specifications of identified commercial VDSs.

##### 3.2.1.9. Passive Sonars—Towed Array Sonar (TAS)

The operation principle of a towed array sonar (TAS) is based on the deployment of very long arrays of hydrophones (tens to hundreds of meters) towed behind a surface vessel or a submarine.

They are mainly used for the detection of large underwater platforms (e.g., submarines). Therefore, they could be used for detecting hostile underwater marine objects of large magnitudes (e.g., submarines).

Table 13 presents the main specifications of identified commercial TASs.

##### 3.2.1.10. Passive/Active Sonars—Hull-Mounted Sonar (HMS)

The hull-mounted sonar (HMS) is used to detect large underwater platforms, such as submarines or large AUVs. They have two sensing principles of operation: passive or active. A passive-sensing HMS is based on detection arrays, spherically or cylindrically distributed. They do not transmit energy but just “listen” to the environment. They are designed using many beams in the horizontal plane. The active-sensing HMS uses cylindrical geometry in order to form a large number of horizontal beams and a few vertical ones. They transmit an acoustic signal to the environment and listen to the backscatter signal received.

Focusing on this case of study, HMS could be used to detect large underwater platforms (e.g., large AUVs) inside the underwater environment where the underwater communication cables are.

Table 14 presents the main characteristics of some identified commercial HMSs.

##### 3.2.1.11. Passive Sensors—Hydrophones

A hydrophone-array mounted platform on the seabed can perform different duties:

- Detect submarines or large AUVs. An example of this is the SOSUS (Sound Surveillance System) network. It was successful used during the Cold War for tracking submarine-radiated noise transmitted through the Sound Fixing and Ranging Channel (SOFAR) channel and for localizing them by triangulation. Interest on SOSUS was lost after the end of the Cold War. More information about SOSUS can be found in [38].
- Detect open circuit divers. Previous research works [39,40] have shown it is possible to detect open circuit divers using hydrophone arrays installed on the seafloor.

Table 15 presents the main characteristics of commercial general-purpose hydrophones that could be used to compose a hydrophone-array for monitoring underwater acoustic noise in order to detect the noise generated by the potential threats (submarines, large AUVs and divers) and to detect them.

##### 3.2.1.12. Dipping Sonar

A dipping sonar is mainly used for detecting large underwater vehicles/platforms, such as submarines or large AUVs. A dipping sonar is an active sonar that is usually dipped from a helicopter at a certain depth, depth optimized based on the knowledge of the underwater environmental conditions.

Since a dipping sonar can detect large underwater vehicles/platforms, a dipping sonar is suitable for the aim of this paper. 

Table 16 presents the main characteristics of some identified commercial dipping sonars.

##### 3.2.1.13. Acoustic Doppler Current Profiler (ADCP)

The acoustic doppler current profiler (ADCP) is used to measure underwater currents. The principle is to process the Doppler shift of echoes from particles (e.g., phytoplankton, zooplankton, bubbles) existing in the water column. These particles are acting as scatterers. Measuring their speed is equivalent to measure the water current.

The motivation for considering an ADCP in this study is its ability to detect changes in the underwater environment along the water column. So an ADCP turns into a sensing capability suitable for detecting potential intruders in the underwater environment where the cables are. 

Table 17 presents the main specifications of some selected commercial ADCPs.

##### 3.2.1.14. Altimeter

Altimeters measure the altitude of an object above the seafloor. They measure range as a function of the two-way signal travel time between the transducer and the seabed (or target). They transmit an acoustic burst from the transducer into a narrow conical-shaped beam. An altimeter is an important sensor in ROVs and AUVs.

Altimeters allow detection of anomalies and changes in the sea bottom along different observation times. This way of operating altimeters may help to identify the presence of intruders in the underwater cable environment or the activity in the sea bottom where the cables are buried.

Table 18 presents the main specifications of identified commercial altimeters.

#### 3.2.2. Magnetometers/Gradiometers Sensors

Typical objects that can be detected by magnetometers are pipelines, underwater communication and power transmission cables, anchors, chains, and UXOs. Detailed information about magnetometer operations can be found in [41]. In general, one ton (1000 Kg) of steel or iron is expected to give a 1 Newton anomaly at 30 m. The amount of distortion falls off as the cube with distance and is linear with mass, so it is possible to detect an object of 100 kg at 15 m or an object of 15 kg at 8 m, or an object of 2 kg at 4 m. These factors are for induced magnetic fields only. Many targets may have permanent magnetic effects or they can be hollow. This would imply changes in detection ranges.

Gradiometers are pairs of magnetometers attached in tandem. The measurement differences between the two magnetometer sensors, divided by the distance separating them, gives the gradient of change of the magnetic flux lines.

Magnetometers on AUVs are used to track underwater communication cables [42]. For the detection, low-frequency currents are applied to the cables’ conductors producing coaxial alternating magnetic field that is measured by the magnetometers. Also, during the installation process of underwater communication cables it is necessary to perform a magnetometer survey in places where the survey route crosses existing cables or to detect ferrous metal objects that could lead to cable damages [7].

Focusing on the aim of this paper, magnetometers can be used to detect anchors or fishing trawls that, as mentioned in Section 2.3, constitute the main cable threats. Hence the importance of Magnetometers or Gradiometers in the construction of underwater cable surveillance networks.

Table 19 presents the main specifications of identified commercial magnetometers/gradiometers.

#### 3.2.3. Optical Sensors

Although both acoustical and optical signals are propagation waves, light waves are electromagnetic waves, while sound waves are pressure waves. This means that light waves do not rely on the existence of a medium to propagate unlike sound waves. Optical signals in general have better resolution than acoustical systems. But their underwater sensing range is significantly shorter due to signal attenuation. Optical cameras maximum range is typically less than 20 m in ideal conditions [43].

The underwater visibility is hindered by scattering and constituents within the medium, and it depends on the availability of natural light. Natural light disappears with increasing depth. Red color disappears at about 5 m depth, orange at 10 m depth, yellow at 20 m depth, and green at 25 m depth. Blue light can still be available at greater depths but the use of artificial lights for photography and other electro-optical (EO) imaging systems is necessary.

When an artificial light source is used, the imaging system performance is reduced by backscattering, as it affects the image contrast. This is especially true for the case when the light source is close to the camera. Several methods are used to address this issue, such as: separating the light source and receiver, range gating, and synchronous scanning between the light, the receivers and the modulated systems, especially in non-line-of sight (NLOS) imagers [44].

The main optical systems used underwater are cameras. The cameras can be video or stills. Table 20 presents the main specifications of some identified commercial video cameras. The cameras can have high-definition properties, making them appropriate for short-range surveillance tasks and scientific observations. There are also low-light-level cameras (usually monochrome) applicable for low-light-level underwater surveying and navigation. Table 21 presents the main specifications of some identified commercial stills cameras. Nowadays, it is common for video cameras to be able to also produce stills images. The advantage of producing stills images is the low energy consumption compared to the video. This makes them suitable to be used in UUGs without compromising UUG endurance.

Despite their limited range (short-range surveillance capability), cameras are valuable tools for the close inspection of the underwater environment where the cables lie.

#### 3.2.4. Oceanographic Sensors

The motivation for measuring oceanographic variables in the underwater environment where the cables are is that any anomaly observed in their values without any obvious-physical explanation is an indication of the presence of a threat/an intruder, such as an underwater platform/vehicle. This is reinforced in the case where more than one, unrelated, oceanographic variable (e.g., salinity, turbidity, pH) simultaneously presents anomalies in their values.

The relevant oceanographic variables for indirectly detecting traces of threats in the underwater environment of an underwater cable are:

- Conductivity, Temperature, Salinity, Pressure and Sound Velocity
  These five oceanographic variables are interrelated. Salinity calculations from conductivity are dominated by the temperature dependence of conductivity. Sound velocity depends on temperature, salinity and hydrostatic pressure. Knowing the sound speed profile is important for the operation of the different kind of sonars studied in this paper. Detection of changes generated by an object/platform/intruder in any of these five oceanographic variables could lead to detect an anomaly in the underwater environment under surveillance. Further knowing the sound speed profile is of paramount importance to many sonar applications to optimize their detection capabilities.
- Turbidity
  Turbidity is the cloudiness or haziness of the sea water caused by suspended particles. It can be an indicator of the presence of an intruder (e.g., excavating the seabed to find a cable, a fishing trawl, and an anchor) that disturbs the underwater environment.
- pH
  The range of seawater pH is typically between 7.5 and 8.4. Any significant change in the pH value of the seawater in the underwater environment where a cable is may lead to the detection of the presence of the underwater object generating such a pH change.

Table 22 presents the main specifications of identified commercial systems that measure the oceanographic variables studied above, which are relevant to survey the underwater environment where communication cables are.

#### 3.2.5. Tables of Acoustic, Magnetic, Optical and Oceanographic Sensors

The main characteristics of the identified commercial sensors and sensing systems are provided in the tables included in this Section 3.2.5. The values are provided according to the manufacturer’s documentation. There is one table for each of the sensors studied in Section 3.2. Some of the identified systems have optional characteristics. The values given here provide indicative parameters and values, according to manufacturer’s specifications. Real performances depend on environmental conditions and cannot conveniently be summarized here.

The tables provide indicative commercial sensors from a limited number of manufacturers. This report is by no means an exhaustive overview of all possible sensors and manufacturers. It has to be noted that the name of each company in the tables appears in the same way shown in the main website of each company. 

The general abbreviations used in the tables are: N/P: Not Provided; N/A: Not Applicable.

## 4. Analysis of Sensors Needed to Secure Underwater Communication Cables in a Multi-Threat Sabotage Scenario

Once analyzed the legal aspects of deploying underwater cables, the national and industrial responsibility, the potential threats existing, and the platform and sensors that could serve to create a surveillance capability, Section 4 focuses on discussing the sensors that should be needed in a multi-threat scenario (case study). Section 4.1 defines the main parameters of the case study. It provides details on the cable parameters (diameter, burial), the underwater environment (range, depth), the monitoring/surveillance zones, the potential threats and the nature of the sabotage actions. Section 4.2 presents proposals on prospective sensors that could be used to monitor the underwater areas of the cables against the identified threats. Section 4.3 discusses integrated solutions (platforms and sensors) for periodic surveillance networks, while Section 4.4 presents ideas for permanent surveillance networks. Finally, Section 4.5 discusses the issue of cable tapping, an important consequence of intentional man-made threats.

Note that the aim of the current Section is to discuss the applicability of sensors and platforms for the case under study based on the specification tables of Section 3 and the public domain knowledge/bibliography. The aim is to present ideas (concepts) on prospective security solutions to secure underwater cables to support decision makers, and not to provide detailed calculations. The actual feasibility of the sensors/platforms systems has to be further assessed in more detail, strongly conditioned by the real risks the surveillance network wants to cover and the available budget.

In specific cases, some calculations are provided in order to use them in the case study. The calculations are only for providing a rough indication on how to proceed and they are by no means accurate representations of the actual platform’s and/or sensor’s capabilities.

### 4.1. Definition of the Scenario

**Figure 6:**
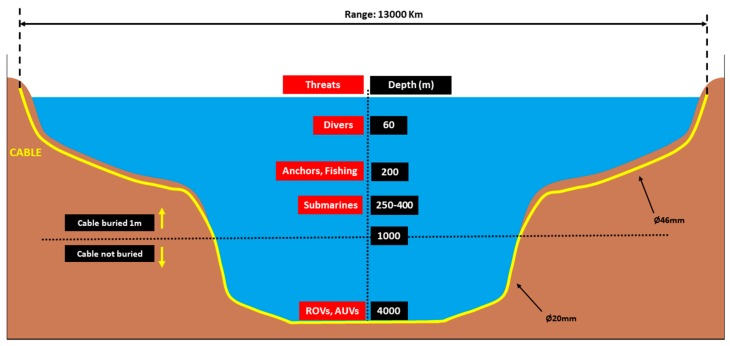
Schematic synthesis of the underwater environment of the multi-threat sabotage scenario. Ø = cable diameter.

A transatlantic cable is considered connecting the USA and Europe in this study. It is a hypothetical case based on realistic parameters [2]. It goes from landing station to landing station. The length of the cable is 13,000 km approx. The maximum water depth encountered is 4000 m approx. Figure 6 presents a schematic synthesis of the environment and the parameters of the multi-threat scenario/case study.

From the analysis of the cable specifications and seabed installation provided in Section 2.1, the cable is expected to be buried from the shore until reaching a water depth of approximately 1000 m. The burial depth is assumed to be 1 m. In deeper waters (>1000 m), the cable is assumed to be laid freely on the seabed. The diameter of the cable is assumed to be 46 mm up to 1000 m water depth and 20 mm for deeper waters. All this is summarized in Figure 6.

The identified potential underwater threats involved are (sorted by risk depth): (a) divers, (b) anchors, (c) fishing trawls, (d) submarines, and e) ROVs and AUVs. Although the anchors and fishing trawls—under normal circumstances—fall into the accidental man-made threats, it is considered in this case study that they can also be used as camouflage for malicious acts (e.g., sabotage). In the case of anchors and fishing trawls, any action will result in cable’s damage or failure. Cable tapping (and cable cut) can be performed by divers and tool handling ROVs and AUVs. The submarines can act as underwater platforms from where ROVs and AUVs can be deployed.

**Figure 7:**
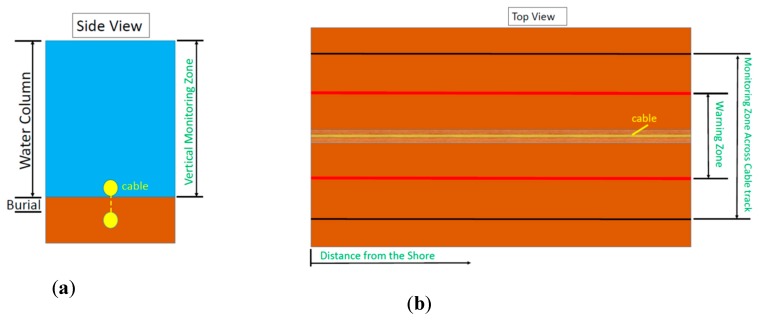
Monitoring areas of underwater cables. (**a**) Vertical monitoring zone, (**b**) horizontal monitoring zones: across cable track and warning zone.

The monitoring/surveillance area for this scenario comprises three parts: (a) a vertical component along the water column, and (b) two horizontal components (on the seabed): one constituting the across-track width of the monitoring zone, and another one constituting the warning zone (narrower and inside the monitoring zone). Any detection inside the warning zone should require immediate action. A schematic representation of these monitoring areas/zones is presented in Figure 7. Table 23 summarizes the main parameters for the multi-threat sabotage scenario under study.

### 4.2. Sensors to Detect Multiple Threats in the Sabotage Scenario under Study

Underwater threats, which are considered in this case study (see Table 23) are listed below, for which recommended sensors are provided in the following subsections:

- Threat group 1—Intentional man-made threats: divers, submarines, ROVs and AUVs.
- Threat group 2—Intentional man-made threats: anchors, fishing trawls.
- Threat group 3—Intentional man-made threats: submarines.

#### 4.2.1. Proposed Sensors for Threat Group 1: Divers, Remotely Operated Vehicles (ROVs) and Autonomous Underwater Vehicles (AUVs)

Divers, ROVS, and AUVs are intruders of similar natures. They are moving underwater bodies which could cause damage with their hands or manipulating arms. Divers often use diver propulsion vehicles (DPVs) to reach their target and perform their mission faster. Their main difference is their operation depth. On the one hand, the maximum operating depth for closed-circuit combat divers is approximately 15 m, while for open-circuit combat divers it extends up to approximately 58 m [49]. On the other hand, ROVs and AUVs can reach very deep waters (thousands of meters). The length of AUVs and ROVs varies. An assumption is made that threats are vehicles of substantial size (size greater than divers) able to have manipulator arms.

The most efficient solution for diver detection, which can also be used for the detection of underwater vehicles (ROVs, AUVs), is the DDS (see Table 11). The sonars operate at approximately 70 kHz having an acoustic coverage of 360° and a maximum detection range that can reach approximately 700 m for closed-circuit divers and almost 1000 m for open-circuit divers. They can be installed along the water column and are routinely used for asset protection in ports, where the vessel traffic is high and the number of detection targets (threats) large. The maximum operating depth of these devices is around 50 m. Therefore, there is a margin of approximately 10 m (from 50 to 60 m water depth) where a typical DSS cannot detect divers.

For water depths greater than 50 m, divers, and also ROVs and AUVs, could be detected using fishery SBES (see Table 5) or MBES (see Table 6). The advantage of these systems is that they can operate from a surface platform and detect an intruder along the whole water column. In the case of fishery SBES, the maximum detection range of a target can be from 270–1100 m for frequencies 200 Hz–18 kHz, respectively. The bottom detection (for monitoring the environment where the cables are laid or buried) can be from 70 m up to 10,000 m for frequencies 710 Hz–12 kHz, respectively. SBES is usually mounted on surface vessels (USVs as well), but they can also be installed on AUVs.

MBES offers high resolution due to its large number of beams with small width. MBES can sweep large corridors across the cable route. These systems have been designed for seabed mapping but their ability to display and log the whole water column along with their high-resolution can make them potential candidates for intruder detection when the beam footprint is small enough to achieve the necessary resolution. The use of MBES backscatter in target detection on the seabed is questionable, so it is better to use its high-resolution bathymetry mode [50]. In this case, the important factors that define its potential bathymetry target resolution are: (a) the sounding solution spacing and (b) the quality of the bottom detection for each solution [50]. As an example, for the maximum operation depth of divers at 58 m, for a MBES with 1° beam width at 20° beam angle, both the across- and along-track resolutions are approximately 1 m. Therefore, divers, as well as ROVs and AUVs, could be detected at this water depth. As depth increases, the beam footprint becomes larger. So for example: at 100 m depth, both resolutions are approximately 2 m; at 200 m depth, both resolutions are approximately 4 m; and at 400 m depth, both resolutions are approximately 8 m.

For water depths greater than the GCS, it is more efficient to detect ROVs and AUVs—if they perform seabed tapping or cable cutting—using high-resolution towed systems, such as SSS (see Table 7) and SAS (see Table 8), or MBES mounted on an AUV (so part of the water column can still be covered). The operating frequency ranges of SSS and SAS are from 75 kHz to 1800 kHz. These systems are by default part of AUVs, so they can be used up to the maximum depth of the sabotage scenario (4000 m). As an example, if a very high frequency operation mode is selected (e.g., 600 kHz), then the resolution is less than 4 cm for a range of 50 m (distance from the seabed). This means that any object of significant size, including a communication cable on the seabed (if not buried), could be detected. OAS (see Table 10) are also a default part of any AUV and could be used for the detection of any underwater asset which falls inside its scanned sector.

Regarding passive systems, it has been shown that open circuit divers can be detected from hydrophone arrays installed on the seafloor [39,40]. An additional advantage of using seabed hydrophone arrays is that they can capture noise from the excavation activity needed for reaching the buried cable. Table 15 shows that the operating depth of hydrophones is about 1700 m. The cable is supposed to be buried up to 1000 m. Therefore, it would be useful to have a hydrophone array along the cable for the buried section.

Since the cable can be reached only through excavation for the first 1000 m of water depth, the threat activity could be indirectly traced by ADCPs (see Table 17) and/or turbidity sensors (see Table 22). ADCPs’ advantage is that they have usually a vertical beam and 4 slanted beams at 20° or 25° and a range that starts from 30 m and can reach up to 1000 m. So ADCPs could cover the whole or at least a large part of the water column covering a conical-shape area around the vertical. Their operation depth can reach up to 6000 m. So, they could be used at any part of the seabed of the multi-threat scenario under study. ADCPs have been used to observe the movement of fishes (e.g., migration speed of herrings) and animals [51]. So, they could also be considered as detection tools for intruders. Turbidity sensors installed on AUVs or UUGs could detect significant changes in the vertical water column due to suspended solids that may occur from any excavation activity.

Underwater cameras (video and still, see Table 20 and Table 21, respectively) could be used on stationary platforms close to the seabed and in shallow waters (maximum: 20 m depth, if the visibility is optimal) for intruder detection. They could also be installed on AUVs or UUGs for monitoring the underwater environment.

Finally, USVs or UUGs having oceanographic sensors (see Table 22) could be used for detecting potential anomalies in the underwater environment due to the presence of ROVs or AUVs.

#### 4.2.2. Proposed Sensors for Threat Group 2: Anchors and Fishing Trawls

Anchors and fishing trawls constitute similar threats. Both harshly invade and disturb the underwater habitat. Fishing trawls cause physical disruption of the seabed through contact of the gear components with the sediment and the resuspension of sediments into the water column in the turbulent wake of the gear [52]. Regarding anchors, an asymmetrical act could happen due to a vessel dropping a heavy anchor and dragging it in an area where underwater communication cables lay [7]. As discussed in Section 2 (Figure 4), this type of threats is prevalent in shallow waters (typical maximum depth of 200 m), normally within the GCS.

Anchors have different dimensions, mainly depending on the vessel that carries it. The study performed in [53] used anchors weighing 8.4 and 11.7 tons, with approximate dimensions of 2.8 × 3.6 m, and with an anchor chain of 60 m. The maximum penetration depth was 0.88 m. Although this is lower than the minimum recommended burial depth (1 m), it is assumed that this depends on anchor (type of ship) and seabed type. Otherwise, the percentage of cable faults due to anchors would not have been too high (see Figure 3). Furthermore, [7] states that for soft to firm sediments, ship anchors may penetrate 2–3 m into the seabed.

Fishing trawls mainly affect the upper layer of the seabed, usually penetrating it around 0.5 m [7]. In the case of the cable not being sufficiently buried, any strike of a trawl would damage it. For example, the impact force of a 1900 kg (weight in water) trawl door, when towed at 2.9 knots is calculated to be about 11 tons [54]. Fishing beam trawls are made of steel and they are up to 12 m long.

Based on the dimensions and properties of the anchors and fishing trawls described above, they could be detected using towed magnetometers (see Table 19). As an example, an anchor of 20 tons could be detected by a magnetometer at a range of 120 m. Their maximum depth rating (2730–6000 m) exceeds by far the required maximum depth of 200 m. Multiple magnetometers could be installed on a pole in order to simultaneously cover larger areas.

As previously discussed, dangerous anchors and fishing trawls are of significant size and weight. As they operate on the seabed, they could be also detected using high-resolution seabed mapping systems, like MBES (see Table 6), SSS (see Table 7) and SAS (see Table 8). As discussed in the previous sub-section, for the MBES, the beam footprint at 200 m water depth, both across- and along-track resolutions, is approximately 4 m. Therefore, it would be able to detect large anchors and fishing trawls that could damage the cable and at the same time monitor the water column for detecting other types of intruders. The towed SSS and SAS systems can operate close to the seabed (where the object of interest is). Operating at very high frequencies (e.g., 1800 kHz) and from a distance of 25 m from the seabed, they could reach a resolution of 0.4 cm, being able to detect the above-mentioned anchors and fishing trawls. A significant advantage of towed systems is that it is possible to install a SSS (or SAS) and a magnetometer in the same tow fish. So the detection accuracy and confidence would significantly increase.

Anchors and fishing trawls heavily disturb the seabed sediment. Therefore, ADCPs (see Table 17) and turbidity sensors (see Table 22) can be used to detect them. As mentioned in the previous Section 4.2.1, ADCPs usually have 5 beams (4 slanted at 20°, and one vertical) and can detect changes in the water current velocities due to suspended particles in a conical shape-area around the water column. ADCPs can be installed either on the seabed or on USVs, AUVs and UUGs, and monitor effectively the underwater environment. Turbidity sensors installed on AUVs or UUGs could also detect the impact of anchors and fishing trawlers on the seabed through sensing the changes in the vertical water column due to suspended solids.

AUVs equipped with underwater cameras (video and still, see Table 20 and Table 21, respectively) could be used to monitor the underwater environment and identify possible intruders that are inside the monitoring zone of the cables.

#### 4.2.3. Proposed Sensors for Threat 3: Submarines

In this case study, it is assumed that the operating depth of submarines is between 250 and 450 m [55]. It is also assumed that they can deploy ROVs [56] and/or AUVs, which could operate up to the maximum depth of 6000 m. Typical submarine length assumed in this study is around 170 m [57]. 

Straightforward sensors for the detection of submarines are those sensors that are already used in anti-submarine warfare (ASW), such as: variable depth sonars (VDS, see Table 12), towed array sonars (TAS, see Table 13), passive/active hull-mounted sonars (HMS, see Table 14), and dipping sonars (DS, see Table 16). VDS uses low frequencies, usually in the range of 20 kHz, but sometimes even lower than 2 kHz. So, they can reach depth ranges up to 6000 m. TAS uses even lower frequency ranges (10–20,000 Hz), being able to reach even larger ranges than VDS. HMS operates in the range frequency of 5–30 kHz. They can reach ranges of 16 km when being operated with low frequencies. Finally, DS can operate in low (10 kHz) or medium (100 kHz) frequencies. They can achieve ranges of 18 km for low frequencies. 

The issue with these sensors (except DS) is their size, requiring large platforms/vessels for their operation. It can be seen in Table 2 that there are USVs now available that can accommodate light towed arrays and dipping sonars. Therefore, it is possible to use these two systems on USVs to monitor the underwater area of the cables against submarine threats.

Submarines could also be detected (due to their large size) by fishery SBES (see Table 5) and MBES (see Table 6) operated from vessels at the sea surface. For example, the beam footprint of an MBES both along- and across- track is about 8.5 m for a depth of 450 m (typical maximum depth submarines can operate). So SBES and MBES could also detect submarines.

As discussed in Section 3.2.1.11, seabed hydrophone arrays (see Table 15) have been used in the past for tracking submarine radiated noise. The re-activation of a surveillance system like SOSUS [38] could, therefore, be an option.

The passage of a submarine through the water produces a wake that changes the oceanographic properties of the water (e.g., temperature, salinity). They are slightly perturbed in comparison with the surrounding water [58,59]. Moreover, various chemicals are introduced into the ocean from engine-based objects, such as small quantities of antifouling paint from the hull, copper and nickel from the piping, which carry coolant water and zinc from sacrificial anodes. In addition, when an object is operating submerged, the by-product hydrogen gas from the oxygen generators is discharged continuously, producing a change of the water properties in the area it crosses. This allows detection of a submarine using sensors measuring the oceanographic properties of the water. These sensors can be mounted on AUVs, UUGs or AUVs. Any simultaneous change in the values of multiple oceanographic variables would mean the presence of a submarine. 

### 4.3. Periodic Surveillance Network (Periodic SN)

A conclusion from the previous section (Section 4.2) is that there are several sensors that could be used for detecting multiple cable threats. A next step is the integration of these sensors with the platforms of Section 3.1 for building a surveillance network. The network could be sorted as: Periodic, if platforms move and perform surveys periodically (e.g., through USVs, AUVs and/or UUGs); or Permanent, if platforms are fixed and perform a constant survey of the underwater environment surrounding the communication cable.

USVs (Table 2) have ranges from 22 to 5329 km. In many cases their range is greater than 1000 km, being sufficient to cover the EEZ. In the scenario described in this paper, docking stations should be available along the 13,000 km cable route for their operation along the whole cable route. USVs could carry most types of acoustic sonar, such as SBES, MBES, SSS, SAS, and OAS. USVs could also be equipped with ADCPs, cameras and oceanographic sensors. Nowadays, USVs can also support acoustic sensors like towed active and passive arrays and dipping sonars. Therefore, USVs constitute platforms that could be used in conjunction with almost any monitoring sensor for enhancing the security of underwater cables. In many cases (e.g., for anchors and fishing trawls), it is necessary to detect any potential threat when entering the monitoring zone and certainly before entering the warning zone (see Figure 7b), as their impact could result in cable damage/failure. Therefore, it is important to monitor any suspect movements of surface vessels in the monitoring zone of the cables. It has to be noted that USVs, apart from underwater sensors, could be equipped with above-water sensors (their study is outside the scope of the current work), such as radars or electro-optical (EO)/infrared (IR) cameras capable of automatically detect, recognize and tracking surface threats (e.g., vessels). In addition, USVs could act as a first line of defense by performing inspection tasks on suspicious vessels. Nowadays, it is also possible to simultaneously command and control (C2) multiple USVs, and integrate them with tethered drones from a single C2 station [60].

AUVs (see Table 3) can be equipped with a large number of monitoring sensors, such as MBES, SSS, SBP, OAS, magnetometers, optical/cameras, and oceanographic sensors. Their main disadvantage is their limited range, which typically goes from 44 to 471 km (average 133 km). This means that their use should be mainly limited to the GCS or for specific-targeted operations in the EEZ or high seas. Docking stations could be available for AUV operations along the whole cable route. AUV advantage is that their operation depth is very high, reaching up to 6000 m. So AUVs could potentially follow bottom topography and survey the cable area at any depth. AUVs can be equipped with both downward and upward pointing sensors in order to capture big parts of the water column and reduce any dead zones. It is also possible to have swarms of AUVs for covering larger areas while performing various tasks. Each AUV can leave temporarily the swarm to fulfill their individual assigned task while the swarm could autonomously reorient itself [61].

Due to UUG long endurance at sea, UUGs are anticipated to be an important key player in networks involving maritime surveillance and monitoring. Their range is very high, starting from 3552 km and reaching up to 11,990 km. However, UUGs (see Table 4) are equipped mainly with Oceanographic sensors, i.e., payloads with very low energy consumption, which cannot be used for direct undersea surveillance. These sensors can sense anomalies that may indicate traces of an intruder but cannot directly detect the intruder. During the last few years, industrial efforts have been made to equip UUGs with additional systems like ADCPs or hydrophones (see Table 4) for passive acoustic monitoring (e.g., detecting marine mammals [62]). Research work has also been made to develop towed arrays to be used by gliders to detect larger targets [62]. Table 4 includes a wave glider that operates on the sea surface. Based on its operation principle, it is a glider, but based on its operational field, it is a USV. Wave gliders can provide long endurance and be equipped with more sensors than UUGs. It has to be noted that UUGs, when operating in saw-tooth profile mode, cannot be used efficiently in conjunction with any acoustic system like SSS, SAS or MBES.

It can be assumed that underwater cables are of similar nature to offshore oil and gas pipelines, although the cables operate in a much more difficult environment. Cable length is tenths of thousands of kilometers longer than that of pipelines, they are laid in much deeper waters, and they face much more unpredictable threats than pipelines. Furthermore, seemingly competing countries that may consider tape or harm each other’s underwater cables, are in many cases cooperating for oil and gas exploitation. Nevertheless, some concepts related to pipeline maintenance may apply to the study announced in this document. Pipelines’ maintenance is mainly preventive and much thought is given on constructing both durable and flexible systems in order to avoid regular short-time monitoring. The external inspection of pipelines involves ROVs and AUVs. This highlights that the concept for the near-future use of AUVs is to make them resident in the underwater environment for periods of months [63]. Whenever there is a need to monitor, inspect or detect something, then ROVs and AUVs can act. This is a concept that can be applicable in the case of monitoring underwater cables. AUVs can be placed in strategically located areas of the seabed along the cable path. Whenever, for example, a suspicious ship is detected by surface sensors inside the monitoring zone of the cable, then AUVs could be activated and sent to monitor the underwater area near the ship for identifying its underwater activity.

### 4.4. Permanent Surveillance Network (Permanent SN)

Permanent SNs can be constructed based on the threat to be detected. It is important to mention that building a Permanent SN represents a one-time investment in instrumentation deployment and infrastructure. The sensors could be installed on non-moving platforms like buoys, moorings and seafloor bottom mounts. Some ideas of potential Permanent SNs for the multi-threat scenario are:

- Arrays of multiple hydrophones (uniformly spaced) could be installed on moorings at various depths to address different threats. The installation could be in shallow waters (50 m) for diver and/or AUV detection. It could also be in deeper waters to detect different threats, similar to SOSUS for the detection of submarines [38]. Passive acoustic monitoring systems are most effective when operating in not noisy multipath environments (e.g., low ship traffic).
- When digging into the seabed for searching a cable or when anchors and fishing trawls are used, the seabed is disturbed. In these cases, an array of ADCPs or turbidity sensors on the seabed could be used to detect changes in the water properties due to suspended particles.
- For diver detection, DDS could be installed at regular intervals along the cable route up to the depth of 50 m. The DDS could be installed on mooring lines all along the water column.
- Underwater cameras could be installed on mooring lines in shallow waters (less than 20 m depth) for the detection of potential intruders (e.g., divers, ROVs, AUVs).
- A very effective solution to detect surface threats is to use buoys equipped with above-water sensors such as radar and/or EO/IR cameras. The buoys can be placed at regular intervals along the borders of the cable monitoring zone (see Figure 7b) to detect and recognize suspicious ships. Then they could send early warnings to C2 reach-back centers for triggering further actions. USVs or AUVs could then be deployed for assessing the exact nature of the threat.
- Nowadays, the idea of using underwater cables as platforms for carrying different types of sensors for environmental monitoring is growing [64,65,66]. Various types of sensors could be installed on the cables for monitoring geo-hazards (e.g., earthquakes, tsunamis). The Geophysical and Oceanographycal-Trans Ocean Cable (GeO-TOC) was the earliest example of a submarine cable-based observatory. It was installed in 1997 midway between Guam and Japan using the retired TPC-1 communication cable [67]. This idea could be extended for enhancing the security of the cables [66] by using them as platforms that carry sensors able to detect possible threats or anomalies in the underwater environment.

The ideas proposed here for creating Permanent SNs could be used to support decision-makers while developing networks to secure underwater communication cables.

### 4.5. Cable Tapping versus Cable Cutting: Potential Solutions to Identify These Consequences

Underwater communication cables are transferring important and sensitive data across the seas and oceans. Therefore, it could be more interesting for an intruder to have access to the data by tapping a cable instead of destroying it. Tape and cable cut detection requires comparison of the seabed, where the cable is buried, at different times in order to identify any changes on the seabed environment. As mentioned in Section 4.1 and shown in Table 23, divers, ROVs and AUVs (with manipulating arms) could tape or cut underwater cables.

For identifying tapping or cutting, an efficient approach is to produce high-quality acoustic images of the seabed using towed SSS or SAS (tow fish or AUVs) close to the seabed. For successful comparison of the DTMs (digital terrain models), it is mandatory to collocate at the time of the survey the bathymetry in order to geo-reference the measurements. SBP could be used in conjunction with the other proposed sonars where the cable is buried. Depending on the frequency used by an SBP (Table 9), it could penetrate the seabed up to 150 m in sandy areas. For the frequency ranges of 4–15 kHz, the penetration would be around 40 m and the vertical resolution around 5 cm.

In case any DTM differences are observed at the positions of the cables, AUVs with underwater still cameras and video cameras could be used for obtaining clearer pictures of the nature of the change and provide this information to the decision makers to assess the situation.

In shallow waters, it could be possible to use also MBES. Regarding the backscatter component of MBES, its utilization to monitor the environmental status of the seafloor by performing repetitive surveys on the same area remains limited. Some methodological techniques of analysis of the differences of backscatter seabed maps of the same area at different times can be found in [68,69].

Monitoring the cable area for tapping or cutting can be connected to the seabed coverage phase, which is an attribute for the search phase. The appropriate sensors for mapping the cable seabed are MBES, SAS and SSS. The swath covered by these systems depends on the water depth. So the limits X1 and X2 of the monitoring and surveillance zones (see Table 23), respectively, cannot be conveniently calculated here as the depth in the case study ranges from very shallow to 4000 m. Therefore, each water depth may require a different coverage regarding the selected systems and the number of them needed. 

The seabed topography is of paramount importance for the efficient operation of AUVs. When the cable’s location and shape are given, the AUV has to navigate to the location, identify the cable, position itself, and then either monitor its status (for friendly AUVs) or cut/tape the cable (for hostile AUVs). At present, this task is not very simple [70], so tapping is more probable to be performed by divers. 

## 5. Conclusions

The main contribution of the ORA study presented in this paper focuses on the analysis of several hundreds of commercial sensors and platforms (to carry them), including the identification and reporting of the most suitable ones. All this was done focusing on the monitoring of the underwater environment where communication cables lay. The sensors identified as most suitable in this case study/scenario use different operating principles, such as: acoustic, magnetic, optical and oceanographic. The platforms identified from this research are: USVs, AUVs, and UUGs. Potential threats were identified, based on recent statistics, and categorized as natural hazards and man-made threats. The study reported in this ORA research paper focuses on intentionally man–made threats. To illustrate this research, a multi-threat case study/scenario was investigated. The water depth in this scenario ranged from shore (some meter depth) to very deep waters (4000 m). The range covered transatlantic distance (13,000 km). The potential threats involved divers, anchors, fishing trawls, submarines, ROVs and AUVs. The applicable sensors for each threat and depth were identified and carefully analyzed, providing useful information to decision-makers on the design of unmanned/autonomous surveillance capabilities. Furthermore, useful information about the integration of the sensors in the platforms was provided.

One of the main conclusions that could be drawn from the reported research is that the cable, as a target, constitutes a very difficult asset to protect. It is not a single-spot target (like a ship). It can be considered as a continuous multi-spot target of thousands of kilometers. This implies target depth largely ranges along the cable route. Consequently, multiple threats of different natures could be encountered.

Specific conclusions regarding the identified sensors:

- Acoustic sensors are the ones commonly used for efficiently monitoring the underwater environment due to their long detection ranges (compared to other sensors). SBES (fishery) and MBES could be mounted on surface vessels and monitor the water column, as well covering the seabed for almost any water depth, if selecting the appropriate frequency. These systems could also be used for target detection in shallow waters or for submarine detection in deeper waters. Towed SAS and SSS could be used for mapping the seabed of the cable location with high resolution and for identifying cable tapping/cutting. When the cable is buried, SBPs could also be used for the same purpose, as they use frequencies that can penetrate the seabed and map its first layers. DDS is the most efficient acoustic sensor for diver and underwater vehicles (AUVs, UUVs, UUGs) detection in shallow waters. ADCP is also a valuable sensor, as it could be installed in any platform, measure turbidity (being generated by the threat) and detect moving objects. One of its main characteristics is that it has multiple slanted beams, so it covers significant areas across the water column. From the point of view of passive systems, towed arrays could be used for the detection of large underwater vehicles. Permanent SNs composed of arrays of hydrophones could be installed on the seafloor to detect underwater noise and threats perturbing the underwater environment (e.g., submarines, AUVs).
- Magnetic sensors (magnetometers) could be used for the detection of possible dangerous ferrous objects. They are very useful for detecting anchors and fishing trawls.
- Optical sensors have short ranges (maximum 20 m). Then underwater video and still cameras could be used for closer inspection of underwater cable areas in shallow waters.
- Oceanographic sensors could be used to detect anomalies in oceanographic variables, which could potentially and indirectly indicate the presence of underwater threats (e.g., submarines, ROVs, AUVs).

From the point of view of the platforms, the main conclusions are:

- USVs could support most of the sensors covered in this study: acoustic, optical and oceanographic. Their range (in most cases) is sufficient to cover at least the EEZ.
- AUVs could also be equipped with a large number of acoustic, optical, and oceanographic sensors. Their main disadvantage is their limited range, which means they could mainly be operated in the GCS or for specific targeted offshore operations. Their advantage is that they could reach depths up to 6000 m.
- UUGs are equipped with low energy consumption payloads, mainly with oceanographic sensors, ADCPs and hydrophones. This limits their detection capabilities (mainly to passive sensing). Their advantage is that they can cover long distances. Then, in many cases, they could be able to survey the cable along its whole route.

An overall conclusion of this research paper is that it is not possible to monitor the environment of the cables with only one type of sensor and platform. A network of them is needed, either depending on the depth or on the threat (size, type). As an example, acoustic sensors could perform detection tasks for early warnings that could trigger the tasking of other systems, such as underwater cameras that would perform recognition tasks. This is why Periodic SNs have a significant value while surveying and monitoring the underwater areas where the cables lay, especially when incorporating different kind of sensing capabilities (acoustic, optical, magnetic and oceanographic).

Future works are identified in this study. Surface platforms equipped with the aforementioned marine sensors and other surface sensors (e.g., radars and/or EO/IR cameras) could play a vital role in the detection and recognition of hostile surface intruders in the cable zone. They could be integrated with underwater SNs (Periodic or Permanent) in future works. The communication network needed for building an effective joint (surface and subsurface) SN (Periodic or Permanent) for enhancing cable security could also be studied in future works.

Finally, highlighting that due to the long lengths of underwater communication cables, it is impossible for a single country to act alone in securing such cables. Therefore, cooperation is necessary for the security and protection of these valuable assets (critical infrastructure).

## Figures and Tables

**Table 1:** Shallow to deep-water (left to right) fiber optic cables’ specifications.

	Double Armor	Single Armor	Single Armor Light	Light-Weight Protected	Light-Weight
Diameter	Ø ~46 mm	Ø ~31 mm	Ø ~28 mm	Ø ~22.5 mm	Ø ~20 mm
Depth (D)	D <500 m	500 m < D < 1000 m	1000 m < D < 1500 m	1500 m < D < 3000 m	D > 3000 m
Burial	yes	maybe	maybe	no	no

**Table 2:** USVs (unmanned surface vehicles) for carrying underwater sensors to survey the underwater environment of communication cables.

Name	Manufacturer	Endurance (h)	Range (km)	Supported Sensors / Capacity (Where Provided)	Speed (knots): Survey–Max
ARCIMS	ATLAS ELEKTRONIK	18	166	MBES, SSS, SAS, ASW (towed active and passive arrays, dipping sonar) / 4 tons	5–40
Sonobot	EvoLogics	10	40	SBES, MBES, SSS / N/P	2.2–6.4
Sounder	KONGSBERG	480	3552	MBES, SBES / 4 kW	4–12
M80	OCEAN*α*	N/P	N/P	MBES, SBES, SSS, OAS, ADCP, / 150 Kg	6–12
APACHE 6	CHCNAV	3	22	MBES / 60 Kg	4–7
C-Enduro	L3HARRIS	720	5329	CAM, CTD, ADCP, MBES, SSS, PAM, ASW (towed array or dipping) / N/P	4–6.5
C-Worker 8	L3HARRIS	168	1243	CTD, PAM, ADCP, MBES, SBP, SSS, vCAM / 3 kW	4–10
Drix	iXblue	168	1243	MBES, SSS, SBP, ADCP, CAM, ASW (towed array light sonar) / N/P	4–14
INSPECTOR 125	ECA Group	40	N/P	SAS, AUV, Ocean / 3 tons	N/P–25

**Table 3:** AUVs (Autonomous Underwater Vehicles) for carrying underwater sensors to survey the underwater environment of communication cables

Name	Manufacturer	Depth Rating (m)	Endurance (h)	Range (km)	Supported Sensors/ Capacity (Where Provided)	Speed (knots): Survey–Max
Bluefin-21	GENERAL DYNAMICS	4500	25	138	SSS, SBP, MBES	3–4.5
Bluefin-9	GENERAL DYNAMICS	200	8	44	SAS, CAM, Ocean	3–6
Remus 100	HYDROID	100	12	66	SSS, Ocean	3–5
Remus 600	HYDROID	600	24	133	SSS, SAS, vCAM, sCAM, SBP, Ocean	3–4
Remus 6000	HYDROID	6000	22	122	ADCP, SSS, Ocean, SSS, MBES, vCAM, sCAM, SBP	3–4.5
Hugin	KONGSBERG	4500	74	410	SAS, MBES, SSS, SBP, Ocean, sCAM	3–6
Hugin superior	KONGSBERG	6000	72	399	SAS, MBES, SBP, Ocean, ADCP, Magnet	3–5.2
Munin	KONGSBERG	1500	24	133	SAS, MBES, SSS, SBP, Ocean, sCAM	3–4.5
Explorer	International Submarine Engineering	6000	85	471	MBES, SSS, SBP, SAS, vCAM, sCAM, Magnet, Ocean	3–2.5
Theseus	International Submarine Engineering	1000	N/P	N/P	Capacity: 550 Kg	4
Gavia	TELEDYNE MARINE	1000	6	33	SSS, SBP, Ocean, ADCP, CAM, Magnet	3–5.5
SeaRaptor	TELEDYNE MARINE	6000	24	133	SBP, MBES, SSS, CAM, Ocean	3–4
SeaCat MK2	ATLAS ELEKTRONIK	600	10	55	SSS, various heads	3–6
Sabertooth	SAAB	1200	14	51	SSS, MBES, SBP, Magnet, manipulator arm	2–5
A9-M	ECA Group	200	20	111	SSS, vCAM, Ocean, SAS	3–5
A18-M	ECA Group	300	21	116	SAS	3
ALISTAR 3000	ECA Group	3000	12	66	vCAM, SSS, SBP	3–4
Seabed AUV	Seabed Technologies	5000	24	133	sCAM, MBES, Ocean	3
MARLIN Mk3	LOCKHEED MARTIN	4000	80	N/P	OAS, SAS, ADCP, SBP, Ocean	N/P–6
Iver4 PW	L3HARRIS	300	N/P	148	N/P	3–5
Echo-voyager	BOEING	3000	months	N/P	Capacity: 8 tons	3–8

**Table 4:** UUGs (Unmanned Underwater Gliders) for carrying underwater sensors to survey the underwater environment of communication cables

Name	Manufacturer	Depth Rating (m)	Endurance (hrs)	Range (km)	Supported Sensors	Speed (knots): Survey–Max
Wave Glider	LIQUID ROBOTICS	N/A	8640	N/P	Cam, ADCP, Ocean, Hydrophone	0.4–2
Slocum G3	TELEDYNE MARINE	1000	12,960	11,990	ADCP, Echosounder, PAM, Hydrophones, Ocean	0.5–2
Seaglider	KONGSBERG	1000	7200	6661	Ocean, ADCP, Echosounder	0.5–N/P
Seaglider C2	KONGSBERG	200	N/P	N/P	Ocean	0.6–2
Seaglider M6	KONGSBERG	6000	N/P	N/P	Ocean	0.4–1
SEAEXPLORER X2	ALSEAMAR	1000	3840	3552	ADCP, Ocean, Echosounder	0.5–1
AutoNaut 7	AutoNaut	N/A	months	N/P	Capacity: 150 Kg	0.5–5

**Table 5:** Acoustic sensor: single-beam echo-sounder (SBES), F = fishery, SW = sweep, WC = water column.

Name	Manufacturer	Frequency (kHz)	Beam width (°)	Detection Range Target (m)	Detection Range Bottom (m)	WC	Depth Rating (m)	Platform
^F^EK60	SIMRAD	18–200	7 × 7	270–1100	550–7000	Yes	N/P	Vessel, AUV [45,46]
EA600	KONGSBERG	12–200 (−710)	7 × 7	280–850	70–10,000	Yes	N/A	Vessel
Ping	BlueRobotics	115	30	N/P	30	Yes	300	USV, AUV
^SW^EA MCU	KONGSBERG	15–200	N/P	N/P	150	Yes	N/P	Vessel

**Table 6:** Acoustic sensor: multi-beam echo-sounder (MBES), WC = water column.

Name	Manufacturer	Frequency (kHz)	Beam Width: Across x Along (°)	Maximum Range for CW (m)	Range Vertical res. (mm)	Max Swath Angle (°)	Max Swath Width (m)	Bathymetry Resolution (cm)	WC	Depth Rating (m)	Platform
Seabat T20-S	TELEDYNE MARINE	200 / 400	2 × 2 / 1 × 1	400 / 225	N/P	165	N/P	6	Yes	6000	AUV
Seabat T50-S	TELEDYNE MARINE	200 / 400	1 × 2 / 0.5 × 1	400 / 225	N/P	165	N/P	6	Yes	6000	AUV
EM2040-04 MKII	KONGSBERG	200 / 300 / 400	0.7 × 1.5 / 0.5 × 1 / 0.4 × 0.7	635 / 480 / 315	10.5	170	920 / 670 / 410	N/P	Yes	6000	AUV
EM712S	KONGSBERG	40–100	1 × 2	1800	N/P	140	3450	N/P	Yes	N/A	Vessel
EM712	KONGSBERG	40–100	0.5 × 0.5	3600	N/P	140	4200	N/P	Yes	N/A	Vessel
EM304	KONGSBERG	26–34	0.5-4 × 0.5-4	8000	N/P	N/P	5.5 × depth	N/P	Yes	N/A	Vessel
Sonic 2020	R2SONIC	200 / 400 / 700	4 × 4 / 2 × 2 / 1 × 1	200	3	130	N/P	N/P	Yes	4000	AUV, ASV
Sonic 2024	R2SONIC	200/450/700	1 × 2 / 0.45 × 0.9 / 0.3 × 0.6	400	3	160	N/P	N/P	Yes	6000	AUV, ASV
Sonic 2026	R2SONIC	100/200/450	2 × 2 / 1 × 1 / 0.45 × 0.45	800	3	160	N/P	N/P	Yes	4000	AUV
WBMS	NORBIT	400 (200–700)	0.9 × 0.9	160	10	210	N/P	N/P	N/P	6000	AUV, ASV

**Table 7:** Acoustic sensor: side-scan sonar (SSS).

Name	Manufacturer	Frequency (kHz)	Beam Angle H (°)	Resolution across (cm)	Max Swath Width (m)	Range/side (Depth below TX) (m)	Bathymetry	Max Depth Rating (m)	Platform
SYSTEM 5900	KLEIN	600	N/P	3.7 (@50m)	N/P	150	N/P	750	Tow fish
2205	EdgeTech	75–1600	N/P	N/P	N/P	1000–35	Yes	6000	AUV, ASV
Solstice	Sonardyne	725–775	0.15	0.1	200	N/P	Yes	200	AUV
UUV-3500	KLEIN	75 / 100 / 400	1 / 0.76 / 0.32	2.4 / 2.4 /1.2	N/P	1500 / 750 / 200	N/P	6000	AUV
Geoswath Plus	KONGSBERG	125 / 250 / 500	0.85 / 0.75 / 0.5	N/P	780 / 390 / 190	200 / 100 / 50	Yes	4000	AUV
ARC Scout MKII	Marine Sonic Technology	150–1800	0.3	0.4–1.5	N/P	500–25	N/P	10,000	AUV
S-150D	SonarTech	100 / 400 / 900 / 1250	1.2 - 0.3	N/P	1000 / 300 / 100 / 60	N/P	N/P	500	Tow fish
Shark-S900U	Icocean	900	0.2	1.2	N/P	75	N/P	1000	USV, AUV

**Table 8:** Acoustic sensor: synthetic aperture sonar (SAS).

Name	Manufacturer	Frequency (kHz)	Image Resolution along/across Track (cm)	Max Range per Side (m)	Bathymetry Resolution vertical/along/across Track (cm)	Depth Rating (m)	Platform
AquaPix INSAS	KRAKEN	337	3.3 / 3	220	10 / 25 / 25	N/P	AUV
ProSAS-60	SL Hydrospheric	60	10 / 10	1200	Yes	6000	tow
HISAS 1030	KONGSBERG	60–120	2 / 2	260	N/P / 5 / 5	N/P	AUV
T-SAS	THALES	N/P	5@150m	150	N/P	N/P	N/P

**Table 9:** Acoustic sensor: sub-bottom profiler (SBP).

Name	Manufacturer	Frequency (kHz)	Range (m)	Vertical Resolution (cm)	Penetration in coarse Sand-Clay (m)	Depth Rating (m)	Platform
2205	EdgeTech	4–24 / 2–16 / 1–10	N/P	4–8 / 6–10 / 15–25	2–40 / 6–80 / 15–150	N/P	AUV, ASV
SeaKing	Tritech	20 / 200	N/P	N/P	N/P	4000	AUV
K-Chirp 3310	KLEIN	5	N/P	12.5	N/P	600	Towfish
Topas PS-40	KONGSBERG	35–45 / 1–10	2000	10	75	N/P	N/P
Topas PS-120	KONGSBERG	70–100 / 2–30	400	5	50	N/P	N/P
Topas PS-18	KONGSBERG	15–21 / 0.5–6	11,000	15	200	N/P	N/P
SES-2000 AUV	Innomar	115 / 4–15	400	5	40	2000	AUV
SES-2000 Standard	Innomar	100 / 4–15	500	5	50	N/P	Vessel

**Table 10:** Acoustic sensor: obstacle avoidance sonar (OAS).

Name	Manufacturer	Frequency (kHz)	Sector Scanned Horizontal (°)	Angular Resolution Horizontal (°)	Range (m)	Range Resolution (mm)	Depth Rating (m)	Platform
Dolphin 6201	MARINE ELECTRONICS	250	120	0.8	200	15	6000	AUV
SA9520	KONGSBERG	70–100	120	1.4	1000	70	N/A	Vessels
NOAS	Sonardyne	70	90	0.3	1500	N/P	N/P	USV, AUV
Seabat F50	TELEDYNE MARINE	200–400	140	N/P	300–600	2.5	6000	USV, AUV
WBMS-FLS	NORBIT	400	150	0.9	250	10	6000	AUV
MRS 900	EchoLogger	900	360	0.1	60	7.5	2000	AUV
Gemini 720is	Tritech	720	N/P	1	120	8	4000	N/P

**Table 11:** Acoustic sensor: diver detection sonar (DDS).

Name	Manufacturer	Frequency (kHz)	Detection Range Radius (m)	Acoustic Cover (°)	Target Bearing Resolution (°)	Target Position (m)	Depth rating (m)	Platform
Sentinel	Sonardyne	70	900 (open)	360	0.14	1 @150m Range	50	Mooring, Seabed, UUV [47]
Aquashield	DSIT	60	700 (closed) / 1000 (open)	360	0.1	0.5	N/P	N/P
Cerberus Mod2	ATLAS ELEKTRONIK	70–130	700 (closed) / 900 (open)	360	1	0.025	50	Seabed, vessel
WG DDSS	WESTMINSTER INTERNATIONAL	N/P	900 (open)	360	N/P	N/P	50	Mooring, Seabed
Echorium	Koc	70	800 (open)	360	N/P	N/P	N/P	N/P

**Table 12:** Acoustic sensor: variable depth sonar (VDS).

Name	Manufacturer	Frequency (kHz)	Coverage Horizontal (°)	Detection Range (m)	Depth Rating (m)	Platform
ST2400	KONGSBERG	22–29	360	6000 (@22kHz)	150	Towed
CAPTAS-4	THALES	<2	360	Up to second oceanic Convergence Zone	230	Towed from platforms > 3000 tons

**Table 13:** Acoustic sensor: passive sonars—towed array sonar (TAS).

Name	Manufacturer	Frequency Range (Hz)	Operating Depth (m)	Total Acoustic Aperture (m)	Platform
TAS	ATLAS ELEKTRONIK	50–10,000	N/P	N/P	tow
KraitArray	sea	10–20,000	300	150	tow USV

**Table 14:** Acoustic sensor: passive/active sonars—hull-mounted sonar (HMS).

Name	Manufacturer	Frequency (kHz)	Sector Transmission Horizontal/Vertical (°)	Bandwidth, Active (Hz)	Bandwidth, Passive (kHz)	Range (m)	Platform
ASO 713/723	ATLAS ELEKTRONIK	6–9	360/ N/P	660 / 3000	2–12 / 2–5 / 1	N/P	Ship, Submarine
SS2030	KONGSBERG	20–30	360 / 60	up to 3000	N/P	16,000	Ship, Submarine
BLACKFISH	DSIT	5–11	N/P	N/P	2–10	N/P	Ship, Submarine

**Table 15:** Acoustic sensor: passive—hydrophones.

Name	Manufacturer	Frequency (Hz)	Sensitivity (dB)	Operating Depth (m)	Platform
AQ-17	TELEDYNE MARINE	5–10	–176	1732	N/P
AQ-18	TELEDYNE MARINE	7–10	–172	1732	N/P
AQ-25 Broadband	TELEDYNE MARINE	2–90,000	–172	1500	N/P
TAH-1	TELEDYNE MARINE	1–40,000	–190.6	1000	Towed array
T-5B	TELEDYNE MARINE	1–5000	–206.6	300	Towed array

**Table 16:** Acoustic sensor: dipping sonar.

Name	Manufacturer	Frequency Range (kHz)	Pulse Bandwidth (kHz)	Range (m)	Transmission Modes H/V (°)	Operating Depth (m)	Platform
SD9500	KONGSBERG	70–110	10	5000	360 / 60	150	Vessel
AN/AQS-13F	L3HARRIS	9.23 / 10 / 10.77	N/P	18,288	N/P	440	Helicopter

**Table 17:** Acoustic sensor: acoustic doppler current profiler (ADCP).

Name	Manufacturer	Frequency (kHz)	Beams	Beam Width (°)	Max Range (m)	Resolution (cm/s)	Cell Size (m)	Number of Depth Cells	Depth Rating (m)	Platform
Ocean Surveyor	TELEDYNE MARINE	38 / 75 / 150	4@30°	2.6	1000 / 700 / 400	N/P	24 / 16 / 8	128	N/A	Marine structure
Workhorse Quartermaster	TELEDYNE MARINE	150	4@20°	4	210–270	0.1	4–24	255	1500	Vessel, Mooring/Bottom
Pinnacle	TELEDYNE MARINE	45	4@20°	N/P	450 / 550	0.5	16 / 32	255	2000	Vessel, Mooring/Bottom, Marine structure
WorkHorse Monitor	TELEDYNE MARINE	300 / 600 / 1200	4@20°	N/P	110 / 50 / 12	0.1	N/P	255	6000	USV-Glider [48]
Signature 1000	NORTEK	1 MHz	1 vertical, 4 slanted @25°	2.9s	30	N/P	0.2–2	200	300	N/P
Signature 250	NORTEK	250	1 vertical, 4 slanted @20°	2.3s, 2.2v	200	0.1	1–8200	200	300	N/P
Signature 55	NORTEK	55/75	3 slanted @20°	4.5 - 5.5	1000 / 600	0.1	5–20	200	1500	N/P
ADP	SonTek	1500 / 1000 / 500 / 250	N/P	N/P	25/35/120/180	0.1	0.25/0.4/1/2	100	500	Mooring, seabed
SeaPROFILER	ROWE	300 / 600 / 1200	4 slanted @20°	2.7/2/1.01	150 / 75 / 30	0.01	N/P	200	6000	N/P
SeaSEVEN	ROWE	300 / 600 / 1200	1 vertical, 3 slanted @20°	2.7/2/1.01	150 / 70 / 30	0.01	N/P	200	6000	N/P
SeaWATCH	ROWE	300 / 600 / 1200	4 slanted @20°	2.7/2/1.01	150 / 75 / 30	0.01	N/P	200	6000	N/P

**Table 18:** Acoustic sensor: altimeter.

Name	Manufacturer	Frequency (kHz)	Beam Angle, Conical (°)	Max Range (m)	Resolution (mm)	Depth Rating (m)	Platform
ISA500	ImpactSubsea	500	6	120	1	6000	ROV, AUV
PA200	Tritech	200	20	100	1	6800	ROV, AUV
PA500	Tritech	500	6	50	1	6800	ROV, AUV
VA 500	VALEPORT	500	3	100	1	6000	ROV, AUV
1007D	KONGSBERG	120 / 200 / 675	15 / 3 / 2.7	600 / 600 / 125	2.4	11,000	ROV, AUV

**Table 19:** Magnetometers and gradiometers.

Name	Manufacturer	Operating Range (nT)	Resolution (nT)	Operating Zones	Detection Range (m)	Depth Rating (m)	Platform
G-882	GEOMETRICS	20,000–100,000	N/P	Restrictions	Anchor 20 tons = 120 m	2730	Tow
SeaSpy 2	Marine Magnetics	18,000–120,000	0.001	No restrictions	N/P	6000	Tow, AUV, Glider
SeaQuest	Marine Magnetics	N/P	0.001	No restrictions	N/P	3000	Tow

**Table 20:** Video cameras.

Name	Manufacturer	Resolution (TVL)	Minimum Illumination (lux)	Focal Length (mm)	Optical Zoom	Digital Zoom	Angle of View D (°)	Platform	Depth Rating (m)
Surveyor-SD	TELEDYNE MARINE	670	0.4	3.5–98	28: 1	12×	72	AUV	6000
Surveyor-WAHD	TELEDYNE MARINE	800	1.4	3.8–38	10: 1	12×	74	AUV	6000
Explorer PRO	TELEDYNE MARINE	570	2 × 10^−5^	N/P	N/P	N/P	103	AUV	6000
Discovery	DWTEK	570	0.05	3.94–46.05	N/P	N/P	51.5	ROV	4000
SS435	SIDUS	570	0.0001	3.8	N/P	N/P	108	ROV	6000
C460	ROS	570	5 × 10^−6^	4	N/P	N/P	77	ROV	6000
OE14-504	imenco	800	0.05	3.8–38	10 ×	N/P	83.3	ROV	4500
OE14-522	imenco	800	2	5.1–51	N/P	N/P	45.1	ROV	4500
OE13-124/125	imenco	576	1 × 10^−6^	4.8	N/P	N/P	86	ROV	4500
OE15-101D	imenco	570	5 × 10^−3^	4–12	N/P	N/P	102	ROV	4500

**Table 21:** Still cameras.

Name	Manufacturer	Image Resolution (MP)	Focal Length (mm)	Angle of View D (o)	Optical Zoom	Platform	Depth Rating (m)
OE14-408E	imenco	10	6.1–30.5	55	N/P	AUV	4500
Tiger Shark	imenco	14	N/P	39	4×	AUV	6000
CT3023	C-Tecnics	20.8	6.7–13	72	N/P	ROV	1000

**Table 22:** Oceanographic sensors, MR = measuring range, Res = resolution.

Name	Manufacturer	Conductivity (mS/cm)		Temperature (°C)		Pressure (Bar)		Sound Velocity (m/s)		Salinity (PSU)		Turbidity (NTU)		pH		Depth (m)
		MR	Res	MR	Res	MR	Res (FS)	MR	Res	MR	Res	MR	Res	MR	Res	
SWiFTplus	VALEPORT	0–80	0.001	–5–35	0.001	10/20	0.001%	1375–1900	0.001	0–42	0.001	0–1000	0.03	N/A	N/A	200
fastCTD	VALEPORT	0–80	0.001	–5–35	0.001	50–600	0.001%	N/A	N/A	N/A	N/A	N/A	N/A	N/A	N/A	6000
rapidPro	VALEPORT	0–80	0.001	–5–35	0.001	100/200	0.001%	N/A	N/A	N/A	N/A	0–6000	N/A	N/A	N/A	N/P
MIDAS	VALEPORT	0–80	0.002	–5–35	0.002	0–600	0.001%	N/A	N/A	N/A	N/A	0–2000	0.002%	1–13	0.01	6000
SVP 70	TELEDYNE MARINE	N/A	N/A	N/A	N/A	N/A	N/A	1350–1800	0.01	N/A	N/A	N/A	N/A	N/A	N/A	6000
RBR maestro	RBR	0–85	0.001	–5–35	0.00005	N/A	N/A	N/A	N/A	N/A	N/A	N/A	N/A	N/A	N/A	6000
RBR solo	RBR	N/A	N/A	–5–35	0.00005	N/A	N/A	N/A	N/A	N/A	N/A	N/A	N/A	N/A	N/A	1700
4319	AANDERAA	0–75	0.002	–5–40	0.01	N/A	N/A	N/A	N/A	N/A	N/A	N/A	N/A	N/A	N/A	6000
4117	AANDERAA	N/A	N/A	0–36	0.001	600	0.0001%	N/A	N/A	N/A	N/A	N/A	N/A	N/A	N/A	N/P
Hyperion	VALEPORT	N/A	N/A	N/A	N/A	N/A	N/A	N/A	N/A	N/A	N/A	0–1000	0.03	N/A	N/A	6000
MIDAS	VALEPORT	N/A	N/A	N/A	N/A	N/A	N/A	N/A	N/A	N/A	N/A	0–2000	N/A	N/A	N/A	N/P
4112	AANDERAA	N/A	N/A	N/A	N/A	N/A	N/A	N/A	N/A	N/A	N/A	0–500	N/A	N/A	N/A	6000
XCX-CND-RA090	AML OCEANOGRAPHIC	0–90	0.001	N/A	N/A	N/A	N/A	N/A	N/A	N/A	N/A	N/A	N/A	N/A	N/A	6000
XCH-SV-0520	AML OCEANOGRAPHIC	N/A	N/A	N/A	N/A	N/A	N/A	500–2000	0.001	N/A	N/A	N/A	N/A	N/A	N/A	6000
XCH-PRS-10000	AML OCEANOGRAPHIC	N/A	N/A	N/A	N/A	0–1000	0.02%	N/A	N/A	N/A	N/A	N/A	N/A	N/A	N/A	10,000
XCH-TMP-n545	AML OCEANOGRAPHIC	N/A	N/A	–5–45	0.001	N/A	N/A	N/A	N/A	N/A	N/A	N/A	N/A	N/A	N/A	6000
XCH-TRB-3000	AML OCEANOGRAPHIC	N/A	N/A	N/A	N/A	N/A	N/A	N/A	N/A	N/A	N/A	0–3000	0.1	N/A	N/A	500
OCEAN SEVEN 316	IDRONAUT	0–70	0.0003	–3–50	0.0002	0–100	0.002%	N/A	N/A	N/A	N/A	N/A	N/A	0-14	0.001	700
OCEAN SEVEN 304	IDRONAUT	0–90	0.0003	–5–35	0.0001	0–700	0.002%	N/A	N/A	N/A	N/A	0.03–750	0.5	N/A	N/A	700

**Table 23:** Multi-threat sabotage scenario characteristics.

FACTS			THREATS		DESIGN REQUIREMENTS
Environment	Cable	Maritime Zones	Underwater Threats	Acts	Monitoring / Surveillance Zones
Water Depth (D): 0–4000 m Cable length: 13,000 km Exact route of the cable: known	Diameter: ~46 mm for D <= 1000 m ~20 mm for D > 1000 m Burial Depth: 1 m	TW: 0–22 km CZ: 22–44 km LCS/EEZ: 0–350 km High Seas: >350 km	Divers Anchors Fishing trawls Submarines ROVs AUVs	Destroy Tape	Vertical: Whole WC Horizontal–Across Cable track: X1 m Horizontal–Warning: X2 m
